# Polar lakes may act as ecological islands to aquatic protists

**DOI:** 10.1111/j.1365-294X.2012.05596.x

**Published:** 2012-07

**Authors:** K RENGEFORS, R LOGARES, J LAYBOURN-PARRY

**Affiliations:** * Department of Biology, Lund UniversityEcology Building, SE-22362 Lund, Sweden; † Institute of Marine Sciences, CMIMA, (ICM) CSIC, Passeig Marítim de la Barceloneta37-49, ES-08003, Barcelona, Spain; ‡ Bristol Glaciology Centre, School of Geographical Sciences, University of BristolBristol BS8 1SS, UK

**Keywords:** Antarctica, biogeography, dinoflagellates, genetic diversity, population genetics, protists

## Abstract

A fundamental question in ecology is whether microorganisms follow the same patterns as multicellular organisms when it comes to population structure and levels of genetic diversity. Enormous population sizes, predominately asexual reproduction and presumably high dispersal because of small body size could have profound implications on their genetic diversity and population structure. Here, we have analysed the population genetic structure in a lake-dwelling microbial eukaryote (dinoflagellate) and tested the hypothesis that there is population genetic differentiation among nearby lake subpopulations. This dinoflagellate occurs in the marine-derived saline lakes of the Vestfold Hills, Antarctica, which are ice-covered most of the year. Clonal strains were isolated from four different lakes and were genotyped using amplified fragment length polymorphism (AFLP). Our results show high genetic differentiation among lake populations despite their close geographic proximity (<9 km). Moreover, genotype diversity was high within populations. Gene flow in this system is clearly limited, either because of physical or biological barriers. Our results discard the null hypothesis that there is free gene flow among protist lake populations. Instead, limnetic protist populations may differentiate genetically, and lakes act as ecological islands even on the microbial scale.

## Introduction

Eukaryotic microorganisms (protists) are a very large and diverse group of organisms that are comparatively little studied within ecology and evolutionary ecology (e.g. Gerstein & Moore 2011). Protists generally have enormous population sizes, small body size and high reproductive rates (which are partially or entirely asexual). These features probably have profound implications for both intraspecific genetic diversity and population structure (Mes 2008). Protists are assumed to have unlimited dispersal and gene flow and should thus not display any population genetic structure as these would be erased by free gene flow. On the other hand, different species may have different abilities to survive dispersal and to colonize new habitats (e.g. Hughes Martiny *et al.* 2006), resulting in dispersal limitation and potential genetic differentiation. Thus, there is a need for empirical data to understand if and how microbial species differ from larger organisms in terms of genetic diversity and population structure.

Population genetic theory predicts high genetic diversity in microbial populations because of their population size, which has been confirmed by population genetic simulations (Mes 2008). In contrast, genetic diversity can be expected to be low because of clonal reproduction, which is prevalent among microorganisms. Within photosynthetic protists (phytoplankton), there is a growing body of evidence that there is high intraspecific genetic diversity. In marine (Rynearson & Armbrust 2004; Nagai *et al.* 2007) and limnetic populations (Beszteri *et al.* 2007; Logares *et al.* 2009) of diatoms and dinoflagellates, genetic diversity was shown to be very high and clonality low. Phytoplankton ecologists had previously assumed that genetic diversity in aquatic protists should be relatively low because they mostly reproduce asexually and because it was believed that populations were dominated by a few successful strains (Lehman *et al.* 1975). However, among mainly asexual species, a wealth of variation occurs because of mutations and rare (or past) sexual events (e.g. Bengtsson 2003). Further, Bengtsson (2003) showed that predominately asexual species started by sexually produced propagules (e.g. dinoflagellate resting cysts) can maintain initial genotypic variation for an extended time before any effect of clonality can be detected. In contrast, simulations suggest that with mainly asexual reproduction, there will be high diversity at single loci, with fewer multilocus genotypes (more clonality) compared to sexually reproducing species (Balloux *et al.* 2003). However, a few sexual events are sufficient to remove the effect of larger variation within individuals than between (Balloux *et al.* 2003; Bengtsson 2003). Thus, protists populations with yearly sexual events should not differ from sexually reproducing species, although empirical data are still largely lacking.

Genetic differentiation of planktonic populations is not yet well understood. Early investigations using allozymes showed high genetic differentiation within a species of marine and freshwater phytoplankton, respectively (Gallagher 1980; Hayhome *et al.* 1987). Presumably because of a lack of more refined molecular tools, further investigations into the topic did not pick up speed until the first decade of the twenty-first century. A number of studies have emerged recently, but the relationship between genetic differentiation and population connectivity is still not clear. In the sea, on the one hand, there is evidence for largely unstructured populations, as in the diatom *Pseudo-nitzschia pungens* that spans across a 100 km region of the North Sea (Evans *et al.* 2005). On the other hand, there are studies showing genetically differentiated neighbouring marine populations despite dispersal potential through currents (Evans *et al.* 2004; Rynearson & Armbrust 2004; Adams *et al.* 2009; Godhe & Härnström 2010). In addition, Nagai *et al.* (2007) found increased genetic isolation with geographic distance in a marine dinoflagellate species. Data from lake-dwelling phytoplankton are more limited. Kim *et al.* (2004) found genetically distinct populations of a freshwater dinoflagellate in lakes separated by only a few hundred metres. However, as these populations differed with as much as 8.9% in their ITS rDNA nucleotide sequences, it remains inconclusive whether they are really the same species in accordance with the 5% level suggested by Litaker *et al.* (2007). In another freshwater dinoflagellate species, there is evidence of different genetic populations inhabiting the same lake and which correspond to different year classes (Logares *et al.* 2009).

**Fig. 1:**
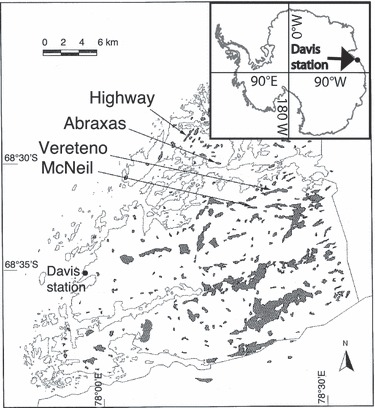
Map of Vestfold Hills showing location of study lakes and Davis Station.

The Vestfold Hills lake area in Antarctica offers a model system for studying the population genetics of protists (Fig. 1). These hydrologically unconnected, marine-derived lakes provide a natural laboratory (Laybourn-Parry & Pearce 2007). The lakes were isolated from the sea through isostatic rebound following deglaciation periods and changes in sea level (e.g. Zwartz *et al.* 1998; Gibson *et al.* 2009). Owing to variation in the amount of evaporation, meltwater and marine ingress, different lakes have developed different salinities. Within the Vestfold Hills lake system, there is opportunity for potential dispersal (either from wind or birds). Although the lakes are ice-covered for most of the year, they are usually ice free for 1–2 months during the summer. Antarctica is little influenced by transport of protists from the other continents (Boenigk *et al.* 2006), and dispersal by anthropogenic processes (boats, fishing gear etc.) is close to negligible because strict protocols are in place to prevent cross-contamination during scientific investigations. The most widespread dinoflagellate species in the brackish and saline Vestfold lake communities is *Scrippsiella* aff*. hangoei*, which is closely related to the Baltic cold-water species *Scrippsiella hangoei* (Rengefors *et al.* 2008). *Scrippsiella* aff. *hangoei* is morphologically identical to the *S. hangoei* in the Baltic Sea, but differs by a few base pairs in the 18S, LSU and ITS rRNA as well as the mitochondrial cytochrome b regions (Logares *et al.* 2008; Rengefors *et al.* 2008). All *S.* aff*. hangoei* populations in the Antarctic lakes are morphologically indistinguishable and have identical ITS sequences, which is evidence that they belong to the same species (Logares *et al.* 2009). DNA fingerprinting analyses using amplified fragment length polymorphism (AFLP) from nine strains originating from four different lakes hinted that strains from the same lake cluster together, although the clusters were not highly supported (Logares *et al.* 2008, 2009).

Here, we have analysed the population genetic structure and differentiation in the photosynthetic dinoflagellate *S.* aff. *hangoei* in four neighbouring lakes within an area with a 10-km diameter. By genotyping between 18 and 38 (haploid) strains per lake on 379 AFLP loci, we were able to calculate the necessary population genetics statistics to make inferences regarding genetic structure and gene flow. We specifically tested the hypothesis that there is population genetic structure among nearby lake populations of dinoflagellates. The null hypothesis was no genetic differentiation because of unlimited gene flow. The alternative hypothesis was that population genetic differentiation would be found among the lakes.

## Materials and methods

### Sampling strategy

Dinoflagellates were collected from four different lakes in the Vestfold Hills area (68°S, 78°E), Eastern Antarctica during the Antarctic summer of 2009. The lakes sampled range in salinity from 5 to 18 (4–18 mS/cm, Table 1) and are all found within 10 km from each other. They include Lake Abraxas, Highway Lake, Lake Vereteno and McNeil Lake (Fig. 1, Table 1). Plankton net tow samples were taken using 10 μm plankton nets.

**Table 1:** Summary of lake data

Lake	Coordinates	Conductivity* (mS/cm)	Chlorophylla† (μg/L)	Altitude (∼m a.s.l)	Area‡ (km^2^)	Max depth (m)	Age (years before present)
Abraxas	68°29′20″S 78°17′13″E	16.3–18.1	0.07–0.2	13	0.063	24 (17)§	>20 000¶
Highway	68°27′47″S 78°13′24″E	6.0	1.2–1.8	8	0.16	∼10	4300–5200**
McNeil	68°31′40″S 78°21′44″E	11.5–11.8	0.2	26	0.071	∼8*	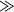 *20 000*
Vereteno	68°30′54″S 78°24′51″E	4.3–4.5	0.22–0.35	0	0.367	25	*2500*

Data available from maps published by the Antartic Australian Division or on own measurements unless otherwise specified. Values in italics are estimated based on papers referred to in ¶ and **. Max depth in parenthesis indicates level of chemocline in meromictic lakes.

* Perriss *et al.* (1995).

† Laybourn-Parry *et al.* (2002); Perriss & Laybourn-Parry (1997); Perriss *et al.* (1995).

‡ Calculated from map.

§ Gibson (1999).

¶ Gibson *et al.* (2009).

** Zwartz *et al.* (1998).

Single cells of *Scrippsiella* aff. *hangoei* [previously identified in Rengefors *et al.* (2008)] were isolated from tow samples using microcapillary pipettes. Cells were placed in half-strength f/2 medium (Guillard & Lorenzen 1972) based on 0.2 μm filtered lake water from each respective lake. Approximately 50 cells were isolated per lake to have a minimum of 20 individuals per population at the end. Strains were subsequently grown at 4 °C, 12:12 light/dark cycle and with light conditions of 20 μmol photons/m^2^/s. Strains that showed positive growth and that were not contaminated by other protists or cyanobacteria were subsequently slowly acclimated to full strength f/2 medium based on seawater from the Öresund region of the Baltic Sea. All cultures were again checked microscopically to ascertain that they belonged to *S.* aff. *hangoei*. The filtered seawater was diluted with deionized MilliQ-water (Millipore Corp., Bedford, MA, USA) to a salinity of approximately 6–7 to mimic the salinity in the Antarctic lakes. Cultures were harvested when a total of 50 mL of exponentially growing culture was available. Cells were centrifuged at 500 ***g*** for 10 min, the supernatant was removed, and the pellet was stored at −80 °C until extraction.

### DNA extraction, AFLP procedure and marker scoring

DNA was extracted using a CTAB protocol based on Dempster *et al.* (1999). Only samples of high DNA quality, that is, with a 260/280 ratio of 2.0, were used for downstream analyses. The DNA samples were stored at −80 °C until genotyping.

All strains were genotyped by AFLP using a fluorescein protocol based on (Vos *et al.* 1995) with five of the primer combinations (A, B, C, D and F) utilized in Logares *et al.* (2009). For the full protocol see Logares *et al.* (2009). AFLP fragments were analysed using a capillary sequencer with a 1000-bp MapMarker. Twelve samples were run in duplicates from separate extractions and including a set of negatives. Peak heights were calculated and binned in GeneMapper and transferred to AFLPScore (Whitlock *et al.* 2008) for normalizing, scoring and error rate calculations. AFLP-dat (Elrich 2006) was used to transfer input files.

**Fig. 2:**
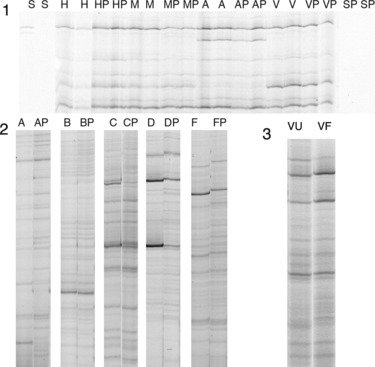
Tests of the potential effect of bacteria on dinoflagellate amplified fragment length polymorphism (AFLP) band patterns are shown. (1) The gel image shows AFLP bands from strains prepared according to standard procedure as well as strains treated with penicillin (P) and supernatant containing bacteria but not dinoflagellate cells from the Highway strain. Four different dinoflagellate strains (one from each lake) and one primer pair combination are shown. S = supernatant (from Highway strain), H = strain from Highway, A = strain from Abraxas, V = strain from Vereteno, M = strain from McNeil, P = penicillin treatment). Note that there are no bands present in the nontreated samples that are missing in the penicillin treatment. Also note that AFLP bands found in supernatant are not responsible for differences among strains. (2) Shows the band patterns of the Highway strains with and without penicillin (P) treatment for five primer pairs (A, B, C, D and F). (3) Shows AFLP pattern of a Vereteno strain harvested by centrifugation (unfiltered) (U) and filtered (to remove bacteria) prior to DNA extraction. Note that there are no differences between the harvesting methods suggesting an effect of bacterial DNA.

Dinoflagellates typically do not grow well without the presence of bacteria, which is why our cultures were nonaxenic (not bacteria-free). We checked that bacterial DNA did not affect the downstream analyses by performing three different tests. First, we compared AFLP band patterns from cells isolated by centrifugation as above (unfiltered), and cells harvested by filtering through a 10-μm nylon mesh to remove bacteria but retain dinoflagellate cells (filtered). In a second test, we applied an antibiotic procedure to reduce bacteria in the dinoflagellate cultures. Four strains (one from each lake) were split into two flasks during exponential growth. One flask was treated with 0.2 mg/mL penicillin G potassium salt (Fluka). Four days later the cultures were harvested. The untreated sample was harvested according to the standard procedure and the other by filtering through a 10-μm nylon mesh to remove any remaining bacteria, followed by centrifugation. The supernatant (collected after centrifugation) from both the untreated and penicillin-treated strains was analysed to detect any bands caused by bacteria. DNA extractions and AFLP analyses were performed as above, except that AFLP fragments were analysed visually using acrylamide gel electrophoresis (as in Logares *et al.* (2009)). Presence of bacteria was checked by microscopical inspection using the DAPI-staining method (Kemp *et al.* 1993) and showed that bacterial number was reduced by 70–75% in the penicillin treatments. The resulting AFLP band patterns showed that there was no difference in band patterns between filtered and unfiltered samples and between penicillin-treated and filtered samples compared to untreated strains (Fig. 2). Moreover, AFLP on bacteria only (supernatant from centrifugation) showed no bands that differentiated strains (Fig. 2).

### Population data analyses

Population structure was analysed using structure 2.3.3. (Pritchard *et al.* 2000). The data set containing 379 loci was entered as haploid and with no prior information on population identity. The priors included the two ancestry models admixture and mixture, in combination with the two allele frequency models (correlated and independent), resulting in four prior settings in total. For each setting, *K* values were set to range from one to eight populations, a burn-in of 20 000 and a run-length of 50 000 steps. Five iterations were made for each setting. The best *K* (number of populations) was determined manually by choosing the lowest *K* where posterior probabilities (log Pr(*X*/*K*)) values reached a plateau.

The number of haplotypes was calculated using Arlequin v 3.5 (Excoffier *et al.* 2005). The ratio of distinct genotypes was calculated by dividing the number of unique genotypes (*G*) divided by the total number (*N*) of strains analysed (*G*/*N*).

Arlequin v 3.5 (Excoffier *et al.* 2005) was used to calculate pairwise genetic differentiation (*F*_ST_) between lake populations, and amova to calculate global genetic differentiation (Global *F*_ST_), genetic differentiation between groups (*F*_CT_) and within groups (*F*_SC_). AFLP-survey v 1.0 was used to determine the number and percentage of polymorphic loci and Nei’s gene diversity (H) within populations (Vekemans *et al.* 2002). A control of population size was carried out by randomly choosing 14 strains from each population (75% of smallest population) and recalculating H.

Arlequin 3.5 was also used to detect loci under selection based on the population inference using the hierarchical island model. Population identity was based on the results from structure, and the AFLP data were coded as RFLP haplotypes. The number of simulations was set to 50 000. Loci with *P* ≤ 0.01 were defined as outliers.

## Results

Isolation and cultivation of *Scrippsiella* aff. *hangoei* strains were successful from four lakes in the Vestfold Hills: Highway Lake, Lake Abraxas, Vereteno Lake and McNeil Lake (Fig. 1, Table 1). Of all the isolations, a total of 108 strains survived (*c.* 50%), distributed among the four lakes. The AFLP analyses yielded 379 AFLP loci after normalization of data based on a 5% Bayesian error rate in AFLPScore. One hundred per cent unique genotypes were identified, suggesting that none of the strains belonged to the same clonal lineage. The percentage of polymorphic loci varied between 17.4% and 28.2% within each of the lake populations (Table 2). The highest number of variable loci was found in Highway, which is also the lake with the highest number of strains. Nei’s gene diversity (H) varied between 0.053 in the McNeil population and 0.086 in Highway. Recalculation of H using 14 random strains from each lake showed the same pattern (Table 2).

**Table 2:** Number of amplified fragment length polymorphism loci, polymorphic loci and Nei’s gene diversity (H) from AFLPSurv

Lake	No. of strains	No. of loci	No. of variable loci	% Polymorphic loci (PPL)	Nei’s gene diversity (H)	(H) for 14 strains
Abraxas	22	379	66	17.4	0.066	0.069
Highway	38	379	107	28.2	0.086	0.094
McNeil	18	379	86	22.7	0.053	0.053
Vereteno	30	379	86	22.7	0.078	0.077

Genetic differentiation between the lake populations was measured by calculating *F*_ST_ values. Global *F*_ST_ was estimated to be 0.245 using AFLPSurv. Pairwise genetic differentiation (*F*_ST_) between lakes was moderate to high ranging between 0.117 and 0.376 (Table 3). All *F*_ST_ values were highly significant (*P* < 0.0001). The highest *F*_ST_ values were found in the pairwise comparisons with McNeil, all of these being around 0.3.

**Table 3:** Pairwise genetic differentiation (*F*_ST_) below the diagonal and minimum geographic distance (km) between lakes above the diagonal in italics. Distances calculated from maps from the Australian Antarctic Division. All *F*_ST_ values are significant at the *P* < 0.0001 level

Lake	Abraxas	Highway	McNeil	Vereteno
Abraxas	–	*3.5*	*4.9*	*4.5*
Highway	0.147	–	*8.75*	*8.75*
McNeil	0.312	0.263	–	*1.3*
Vereteno	0.169	0.117	0.376	–

**Fig. 3:**
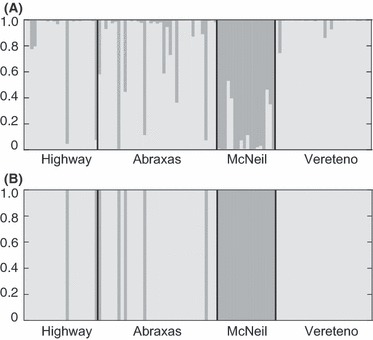
Population analyses results from structure shown as bar plots. Only the models showing the highest likelihood (*K* = 2) are shown. Bars signify individual strains, where *y*-axis shows proportion assignment to populations, and *x*-axis shows original lake population that the strains originated from. (A) Bar plot of structure analyses using a model allowing admixture and independent alleles (corresponding to sexually reproducing populations), with *K* (numbers of populations identified) = 2; designated as light grey and dark grey. (B) Bar plot of structure analyses using a model not allowing admixture and correlated alleles (corresponding to an entirely asexual population), with *K* (numbers of populations identified) = 2; designated as light grey and dark grey.

Genetic structure of the populations was investigated using the software structure. All the ancestry and allele frequency models yielded the highest likelihood for a model with two genetic populations (*K* = 2), regardless of whether admixture (sexual recombination) was allowed (Fig. 3a) or not (Fig. 3b). In all ancestry and allele frequency models, the scenario with *K* = 2 showed a clear pattern of one population consisting mainly of strains from McNeil and the second population consisting of strains from the other three lakes. Further clustering analyses of the population including three lakes did not yield any further subdivision.

Based on the structure results (two populations), we performed an amova. The global *F*_ST_, that is, the genetic differentiation explained by all variation within and among groups or populations, amounted to 0.299. *F*_CT_, which explains the genetic variation between populations, was 0.177, and the variation within groups, *F*_SC_, was 0.148.

To detect potential loci under divergent selection, that is, loci involved in potential local adaptation, the software Arlequin was used to identify loci with higher than expected *F*_ST_ values. Both when the analysis was run with four populations (in accordance with sampling and Arlequin results) and with two populations (based on structure results), a single outlier with a *F*_ST_ higher than the 95% quantile (*P* < 0.05), indicating a locus under divergent selection, was found. A number of loci with low *F*_ST_ values suggesting balancing selection were found significant (*P* < 0.05).

## Discussion

Using population genetic tools and molecular markers, we investigated whether gene flow is unlimited and whether population genetic structure was present in an aquatic free-living protist species (Dinophyceae) found in Antarctic saline lakes. Our results show a clear population structure and high genetic differentiation among lake populations despite the close proximity (<10 km) of the lakes. We also found high genetic diversity within the lakes. Our results and conclusions are discussed in detail later.

### Population genetic structure

The brackish/marine species *Scrippsiella* aff*. hangoei* (hereafter *Scrippsiella*) could potentially belong to one single metapopulation with free gene flow among them. Our data, however, show high genetic differentiation among the four lake populations studied. The range of the values are similar to those found for populations of freshwater diatoms at much larger geographic distances (>100 km) (Evans *et al.* 2009), although it must be noted that those values were measured on microsatellite data and a small data set and are thus not directly comparable. The *F*_ST_ (genetic differentiation) values among the dinoflagellates lake populations are much higher than, for example, freshwater fish, where *F*_ST_ values in the 0.06 range are considered as substantial genetic differentiation (Wang *et al.* 2007). Because of the high and significant *F*_ST_ values, we conclude that genetic differentiation among lake populations is considerable. In comparison, equally high *F*_ST_ values have also been found in some neighbouring marine diatom populations (Rynearson *et al.* 2006; Härnström *et al.* 2011) as well as in a marine dinoflagellate (Alpermann *et al.* 2009). On the other hand, the *F*_ST_ values between sampling sites of the marine dinoflagellate *Oxyrrhis marina* were one magnitude lower (Lowe *et al.* 2010).

Using the Bayesian-based clustering method (structure), two genetically distinct populations were identified in our data set. One population included all the strains from Lake McNeil, and the other consisted mainly of strains from the other three lakes. Regardless of whether a model allowing for sexual recombination (admixture) or for strictly clonal reproduction (no admixture) was used, the same two populations were identified. Whether the *Scrippsiella* Antarctic metapopulation really consists of four separate or two populations is a question of interpretation. While *F*_ST_ values are based on predefined information that strains belong to different lake populations, the structure analyses search for genetic clusters that maximize the likelihood that individual genotypes belong to them. In that sense, the Bayesian method can be considered less biased and more conservative. On the other hand, it is considered that this method does not deal adequately with genetic data from organisms that reproduce asexually to a large extent because they may not be under Hardy–Weinberg equilibrium (Halkett *et al.* 2005). In addition, the amova analyses also support the two-population scenario suggested by structure because the variation between the two structure populations is larger than within the populations. Also, the pairwise comparisons indicate that Lake McNeil is most divergent (highest *F*_ST_ values). By choosing a more conservative interpretation, we conclude that there are at least two genetically distinct populations, of which the McNeil strains form a unique population.

### Dispersal limitation and gene flow

Our analyses suggest that there is limited gene flow among the lakes. Based on the structure analyses (the model where recombination is allowed), we observe that there are about 10% of individuals in the population including lakes Highway, Abraxas, Vereteno, that are mostly (but not entirely) assigned to the McNeil population (Fig. 2). In a model based on clonal reproduction (no recombination allowed), a total of seven individual strains found in Highway and Abaraxas were assigned to the McNeil population. Both models, disregarding whether there is sexual recombination or not, suggest that there is at least some gene flow among the populations, even if it is very limited.

Limited gene flow may be caused by dispersal limitation. The geographic distances between the lakes range between 1 and 9 km (Table 3), but there is no correlation between Nei’s genetic distance and geographic distance, suggesting a lack of isolation with distance at this scale. The lakes are closed basins so all potential dispersal would be through air or by bird vectors. Because ice is absent for 1–2 months per year, the window for dispersal is quite short. Birds such as Skuas, Show Petrels and Penguins are occasionally seen on or around them (personal observations). Moreover, the Vestfold Hills are subject to katabatic winds, and algal spores (Pearce *et al.* 2009) and cysts of dinoflagellates have been observed in air samples (J. Downs & J. Laybourn-Parry, unpublished data). The specific conditions of the area suggest that there may be some physical barriers to dispersal.

### Potential causes of population differentiation

Population genetic differentiation may also be due to biological barriers. For example, cells or cysts may very well disperse, but be either nonviable upon arrival or unable to colonize because of competition with residents. The paradox of reduced gene flow despite high dispersal capacities in aquatic organisms has also been recorded from multicellular animals [cladocerans (Palsson 2000), rotifers (Campillo *et al.* 2009), bryozoans and macrophytes (Charalambidou & Santamaría 2002)] in lentic habitats. A proposed explanation is the Monopolization Hypothesis, which states that large genetic differentiation between well-connected habitats can be explained by rapid population growth after historical founder events, enhanced by a large propagule bank that buffers against new immigrants, and rapid adaptation of resident population to local conditions (De Meester *et al.* 2002).

In the present study, the McNeil population was identified as the most divergent of the four lake populations based on both the *F*_ST_ analyses and the Bayesian clustering analyses. There are several possible explanations for this divergence, either because of physical or biological barriers, or both. Assuming that there are barriers among the lakes, there are two scenarios. The first is that the four lakes have had the same genetic standing stock from the beginning (when isolated from the sea). Differentiation has subsequently resulted because of random mutations and/or natural selection, and by chance, the McNeil population is the one that has diverged most. The other scenario is that the founder population of *Scrippsiella* in McNeil was different from those of the other three lakes, and because of restricted gene flow either because of physical barriers or biological barriers (Monopolization Hypothesis), it has maintained its difference.

Lakes in the Vestfold Hills area are of different ages because of deglaciation history, isostatic rebound and changes in sea water level (Zwartz *et al.* 1998). Highway and Vereteno are both <5000 years old (Table 1). Lake Abraxas existed before of the Holocene marine highstand (∼10 000 bp) and was present (ice free or with thin ice) during the Last Glacial Maximum (20 000 bp) and is thus at least 20 000 years old and perhaps as much as 125 000 years old (Gibson *et al.* 2009). Gibson *et al.* (2009) suggest that the ice cap during the Last Glacial Maximum did not reach far beyond its present point, meaning that the lake was not buried beneath the ice sheet. McNeil is located at an altitude of 26 m indicating that it should be much older than the other lakes. Given the altitude of Lake McNeil, it is likely that its *Scrippsiella* population is older and different than that of the other lakes, thereby supporting scenario number two. However, since the ages of Lakes Abraxas and McNeil are uncertain to date, no definitive interpretation can be made.

### Neutral variation or natural selection?

Given physical or biological barriers to gene flow, natural selection leading to local adaptation could explain part of the differentiation among the lakes. The environment in the lakes differs from the sea, with more variable light, temperature and salinity. Chemical data from previous studies (Roberts & McMinn 1996; Perriss & Laybourn-Parry 1997) suggest that the lakes differ slightly in terms of nutrients, alkalinity, temperature, etc. More importantly, the lakes vary in salinity. Nevertheless, there are no obvious abiotic factors that differentiate McNeil from the other lakes. Abraxas is on average the most saline lake, but the Abraxas population clusters with those from the lower salinity lakes Highway and Vereteno. However, *Scrippsiella* in the Vestfold Hills lakes are found at a wide salinity range (3.5–20 psu) (Rengefors *et al.* 2008), and the closely related (possibly same species) *Scrippsiella hangoei* from the Baltic Sea grows equally well from a salinity of 0 to 30 psu (Logares *et al.* 2007).

In an attempt to determine whether some loci were under selection, we performed an outlier analysis of the AFLP loci. AFLP loci can include both regions in coding and noncoding genes, which is why bands may be connected to genes under selection. Whether we compared all four lake populations or retained the more conservative two populations identified by the genetic structure analyses, we only found one locus that was a significant outlier indicating that it may be under divergent selection. In comparison, a genome scan for loci under selection using 440 AFLP loci in four populations of lake whitefish (*Coregonus* sp.) resulted in a total of 24 loci under divergent selection (Rogers & Bernatchez 2007). Outlier genome scans for selected loci in a lizard showed 3–4% selected loci (Nunes *et al.* 2011). Although a direct comparison is not possible because of differences in genome size, chromosome number and more, our data suggest that selected loci do not play a large part in differentiating the dinoflagellates populations but rather of founder effects. However, a scan of more AFLP loci may have resulted in more selected loci. On the other hand, a number of loci had an *F*_ST_ below expected levels, which indicates balancing selection (Excoffier *et al.* 2009).

### Genetic diversity

Our data confirmed earlier findings that genetic diversity within protist populations is high (e.g. Rynearson & Armbrust 2004; Alpermann *et al.* 2009; Härnström *et al.* 2011). Nei’s gene diversity corresponded to what we had found in an earlier study (Logares *et al.* 2009) using only a few strains per lake. This suggests that reasonable estimates can be made even with small sample sizes. Compared to Nei’s gene diversity in freshwater dinoflagellates, the range here was 3–4× lower (Logares *et al.* 2009). This result could be expected for these polar lakes given that the gene flow is extremely limited and that there is likely a high selection pressure because of varying salinity. Moreover, contrary to earlier assumptions among phytoplankton ecologists (Lehman *et al.* 1975), there is no indication of a dominance of one or only a few genotypes. In the current study, all genotypes were unique (note that dinoflagellates are haploid), which correspond to our previous data on five different dinoflagellates species (Logares *et al.* 2009). The sample size of the latter study was very small (3–4 strains per lake), which is why the current study was necessary to confirm or discard those preliminary patterns. Although the AFLP analysis is robust, it cannot be ruled out that none of the strains are not from the same clone. However, we have previously found that AFLP analyses have been able to detect differences in siblings from the same parental cross in a repeatable manner (Figueroa *et al.* 2009).

The high genotype diversity observed in our study can be explained by population genetic theory, which predicts that even a small amount of sexual reproduction should yield high genotype diversity in populations that otherwise reproduce mainly asexually (Bengtsson 2003). The amount of sexual events in natural populations of dinoflagellates is not known; however, most species with described life cycles have been shown to reproduce sexually in both the laboratory and in the field (e.g. von Stosch 1973). In the field, sexual reproduction is observed in conjunction with resting cyst formation at the end of the bloom (Anderson *et al.* 1983). The life cycle of the Antarctic *Scrippsiella* has not been described, but in the Baltic Sea *Scrippsiella hangoei,* both the formation of asexual and to a lesser extent sexual cysts have been observed (Kremp & Parrow 2006). Antarctic *Scrippsiella* divide approximately every 6 days in culture (Logares *et al.* 2007). In a growth season, about 15–17 generations would be expected (Rengefors *et al.* 2008) and then likely followed by sexual reproduction preceding cyst formation. Given the high genetic diversity in the samples, it is highly likely that the populations occasionally undergo sexual reproduction. Moreover, germinating cysts may originate from different year cohorts given the longevity of cysts (up to 100 years in some species (Ribeiro *et al.* 2011)), and because of bioturbation, and resuspension events of cyst beds (Keafer *et al.* 1992). Germination of different year classes likely helps maintain the high genetic diversity within populations (see De Meester *et al.* 2002; Alpermann *et al.* 2009).

## Conclusions

Our results discard the null hypothesis that there is free gene flow among protist lake populations, despite close geographic proximity. Instead, population genetic analyses showed that gene flow among lakes was restricted and that the four lakes clustered into two or four separate genetic populations. The mechanisms for differentiation remain to be investigated further and environmental effects cannot be ruled out. However, our data suggest that lake age, and thereby isolation time, is a major factor determining population genetic structure, indicating persistent founder effects. To conclude, we suggest that these polar lakes act as ecological islands, not only for macroorganisms but also for microorganisms.

## Data accessibility

AFLP input files: DRYAD entry doi: 10.5061/dryad.87js01tv.
